# Development and application of tools to cost the delivery of environmental health services in healthcare facilities: a financial analysis in urban Malawi

**DOI:** 10.1186/s12913-021-06325-3

**Published:** 2021-04-13

**Authors:** Darcy M. Anderson, Ryan Cronk, Emily Pak, Precious Malima, David Fuente, J. Wren Tracy, Innocent Mofolo, Holystone Kafanikhale, Irving Hoffman, Jamie Bartram

**Affiliations:** 1 The Water Institute, Gillings School of Global Public Health, University of North Carolina at Chapel Hill, Chapel Hill, NC 27599 USA; 2 ICF, Durham, NC 27713 USA; 3 Independent contractor, Lilongwe, Malawi; 4 School of Earth, Ocean and the Environment, University of South Carolina, Columbia, SC USA; 5 UNC Project Malawi, Lilongwe, Malawi; 6 Division of Infectious Diseases, School of Medicine, University of North Carolina at Chapel Hill, Chapel Hill, NC USA; 7 Malawi Ministry of Health and Population, Lilongwe, Malawi; 8 School of Civil Engineering, University of Leeds, Leeds, UK

## Abstract

**Background:**

Environmental health services (EHS) in healthcare facilities (HCFs) are critical for providing a safe, functional healthcare environment, but little is known about their costs. Poor understanding of costs impedes progress towards universal access of EHS in HCFs. We developed frameworks of essential expenses required to provide EHS and conducted an ex-post financial analysis of EHS in a network of medical research and training facilities in Lilongwe, Malawi, serving an estimated 42,000 patients annually through seven outpatient buildings.

**Methods:**

We estimated the cost of providing the following EHS: water, sanitation, hygiene, personal protective equipment use at the point of care, waste management, cleaning, laundry, and vector control. We developed frameworks of essential outputs and inputs for each EHS through review of international guidelines and standards, which we used to identify expenses required for EHS delivery and evaluate the completeness of costs data in our case study. For costing, we use a mixed-methods approach, applying qualitative interviews to understand facility context and review of electronic records to determine costs. We calculated initial costs to establish EHS and annual operations and maintenance.

**Results:**

Available records contained little information on the upfront, capital costs associated with establishing EHS. Annual operations and maintenance totaled USD 220,427 for all EHS across all facilities (USD 5.21 per patient encounter), although costs of many essential inputs were missing from records. Annual operations and maintenance costs were highest for cleaning (USD 69,372) and waste management (USD 46,752).

**Discussion:**

Missing expenses suggests that documented costs are substantial underestimates. Costs to establish services were missing predominantly because purchases pre-dated electronic records. Annual operations and maintenance costs were incomplete primarily because administrative records did not record sufficient detail to disaggregate and attribute expenses.

**Conclusions:**

Electronic health information systems have potential to support efficient data collection. However, we found that existing records systems were decentralized and poorly suited to identify EHS costs. Our research suggests a need to better code and disaggregate EHS expenses to properly leverage records for costing. Frameworks developed in this study are a potential tool to develop more accurate estimates of the cost of providing EHS in HCFs.

**Supplementary Information:**

The online version contains supplementary material available at 10.1186/s12913-021-06325-3.

Contributions to the literature

- We developed frameworks of expenses required to deliver environmental health services in healthcare facilities in low- and middle-income countries that can be used in future studies to assess completeness and accuracy of costs data
- Water, sanitation, hand hygiene and waste management require more upfront expenses for infrastructure than cleaning, personal protective equipment use, and vector control, which require predominantly personnel and consumable supply expenses
- Electronic health information systems have potential to support efficient data collection, but incompleteness and poor disaggregation of environmental expenses impede costing

## Background

A hygienic environment is critical for safe healthcare provision. Two-thirds of healthcare acquired infections (HAIs) are attributable to inadequate hand hygiene of healthcare workers and environmental surface contamination [1]. An estimated 15% of hospitalized patients in low- and middle-income countries (LMICs) acquire an HAI [2], representing billions of dollars spent annually on preventable medical expenses [3].

Environmental health services (EHS) in healthcare facilities (HCFs) reduce the risk of HAIs by reducing transmission of contamination from person-to-person, and person-to-environment and vice versa [4, 5]. EHS protect patients and providers, other workers in the HCF (e.g., waste handlers and cleaners), patient caregivers, and people living in the surrounding communities who may be exposed to waste outputs [5]. Inadequate EHS reduce patient care-seeking [6, 7] and adversely affect healthcare worker satisfaction with the work environment [8, 9]. Integrating EHS programming into the health sector is important to reduce maternal and newborn mortality [10–12] and development of antimicrobial-resistant pathogens [13].

Goal 6 of the Sustainable Development Goals (SDGs) sets targets for achieving universal access to water, sanitation, and hygiene. The World Health Organization (WHO) and UNICEF’s Joint Monitoring Program for Water Supply, Sanitation, and Hygiene (JMP), which monitors progress towards SDG targets 6.1 and 6.2, interprets “universal” to include HCF settings and also monitors waste management and cleaning as EHS. HCFs have been specifically identified as a priority in the United Nations’ Decade for Action to meet the SDGs by 2030 [14, 15]. The JMP’s 2019 report indicates that approximately 26% of HCFs world-wide lack basic access to water; 21% lack sanitation, and 16% lack hand hygiene facilities. Representative JMP data for waste management and cleaning are unavailable [16]. Other sources suggest that 39% of HCFs in LMICs lack handwashing soap, 39% lack adequate infectious waste disposal, and 73% lack sterilization equipment [17].

Adequate financing is critical to improving access [12, 18]. However, the current evidence for understanding the costs of providing EHS in HCFs is weak. A 2020 systematic review describes the quality of available costs evidence as poor and identifies areas for improving evidence through application of models and frameworks designed specifically for costing EHS in HCFs [19].

We describe the first application of a model designed specifically for costing EHS in HCFs in LMICs [5]. We extended the model by developing frameworks of essential outputs and inputs for each EHS, which we used to identify expenses essential for EHS delivery and evaluate the completeness of costs data. We applied this model and frameworks in an ex-post financial analysis of a network of medical research and training facilities in Lilongwe, Malawi, using data from electronic records systems. We present costs estimates and discuss opportunities to improve health information management systems (HMIS) to facilitate EHS costing.

## Methods

### Setting

We collected data in a network of facilities operated by University of North Carolina (UNC) Project Malawi. This is a collaboration between UNC-Chapel Hill and the government-run central hospital in Lilongwe. UNC Project’s primary purpose is to support medical research and training, and it provides free clinical care to patients enrolled in medical research studies, with study populations including patients with HIV/AIDS, malaria, tuberculosis, and cancer. Because patients are research participants, they have longer visits and receive additional laboratory work than the general patient population. In 2018, UNC Project recorded 42,228 patient encounters, 25,849 patient care hours, and 7268 pathology reports.

UNC Project facilities comprise seven buildings, with 70 rooms for outpatient care and a research facility with offices and a 370 square-meter laboratory. The majority of operating space is laboratory, pharmacy, teaching, and administrative. Additional setting description is provided in Additional file 1.

### Framework development

We reviewed selected guidelines for environmental health and infection prevention and control [4, 20–37] to identify essential outputs and inputs for each EHS. We considered outputs essential where they were consistently described across guidelines as important for preventing HAIs or providing a functional and satisfactory work environment. We identified inputs essential to achieve each output and categorized them according to the cost categories identified in Table 1. These inputs represented essential expenses for EHS delivery, and during cost data collection we used these frameworks to identify the costs missing from records.

**Table 1:** Definitions of costs categories. Reproduced from Anderson et al. [5]

Cost category	Definition
Capital hardware	Infrastructure or equipment purchases required to establish services or implement changes to service delivery method, which are not consumed during normal service operation
Capital software	Planning, procurement, and initial training costs associated with establishing new services or implementing changes to service delivery method
Capital maintenance	Expenses required to repair, rehabilitate, or otherwise maintain functionality of capital hardware, including labor costs required for these purposes
Recurrent training	Training required to ensure proper ongoing service provision regardless of changes to service delivery
Consumables	Products and supplies that are consumed during normal operation
Personnel	Labor costs associated with normal operation of a service, including staff benefits
Direct support	Expenses required to supervise and monitor service provision to ensure safety and sustainability that support but do not have direct service outputs, such as auditing or developing management plans
Financing	Loan interest and other fees associated with debt financing
Contracted services	Fees paid to external providers to perform all or part of normal EHS operation, including multiple other cost categories, where expenses cannot be accurately disaggregated into categories above; where fees fall solely within another cost category described above, expenses should be included therein

### Costs data collection

We followed a ten-step process model described by Anderson et al. [5]. Briefly, the model comprises three phases: *Planning* to define the scope of relevant EHS and costs, to understand the facility context, and to develop a data collection plan; *Data collection* to execute the data collection plan and evaluate data quality; and *Synthesis* to calculate costs and disseminate findings. Anderson et al.’s model is designed to be flexible to a variety of contexts and methods. This study employed a mixed-methods approach comprising qualitative interviews to understand facility context and a retrospective records review for costs data collection.

We describe the specific methods used for each step below and in Additional files 1-4. We adhere to the Consolidated Health Economics Evaluations Reporting Standards [38], the checklist for which is included in Additional file 5.

#### Step 1: define costing purpose

We assessed initial costs to establish services and subsequent costs for operations and maintenance. We identified UNC Project as our setting because they provided all EHS outputs that we identified as essential and had electronic records available. We present costs at the network level for EHS across all facilities within UNC Project, as records of disbursement of resources across individual facilities within the network were not available. Where costs were unavailable through records, as was the case for most capital costs, we describe the reasons for missingness and propose good practices for collecting this information in future studies.

#### Step 2: identify relevant EHS

We considered the following as EHS, based on the WHO’s *Essential Environmental Standards in Healthcare Facilities* [4]: water, sanitation, hand hygiene, personal protective equipment use at the point of care (hereafter “PPE”), waste management, surface and medical device cleaning and disinfection (hereafter “cleaning”), laundry, and vector control. We excluded services that are not applicable to all facilities (e.g., food hygiene) or predominantly achieved through building design (e.g., drainage, ventilation, avoiding over-crowding).

#### Step 3: define the scope of relevant costs

We examined costs from the perspective of the HCF network and captured only the expenses documented in electronic records systems. We did not consider economic costs or financial costs incurred outside the HCF network. We defined the timeframe of eligible costs as installation and subsequent annual operations and maintenance. We defined eligible types of expenses as any expense falling into one of the nine categories described in Table 1.

#### Step 4: collect non-costs contextual data

We collected contextual data on the resources required to deliver each EHS and the quantity and quality of EHS in network facilities through interviews with facility staff, including the facility director, administrative officers, head nurse, and staff from the accounting and procurement departments. Interview guides were designed to capture information on modes of EHS provision, resources used, and any challenges with service adequacy or safety. A list of the job titles of participants and the interview guide are provided in Additional file 2.

We assessed EHS quality by reviewing interview recordings and noting instances where interviewees commented on the safety, reliability, or sustainability of services. We collected information on the number and purpose of clinical and non-clinical rooms in each facility and the number and type of staff employed. Information on patient volume was gathered from internal annual reports. Descriptions of EHS resource inputs, quality, and quantity are provided in Additional file 1.

#### Step 5: develop a costing plan

We pilot tested our costing tools and protocol in three out-of-network HCFs (two private clinics and one large central government hospital) prior to data collection at UNC Project. We pilot tested a survey instrument designed to conduct bottom-up costing but found that staff recall was insufficient. Through subsequent discussions with facility administrators, we identified retrospective records review as the best data collection strategy based on accessibility of electronic records and level of detail recorded.

#### Step 6: identify data sources

We identified departments responsible for purchasing resources identified in our costing frameworks, which comprised the laboratory, pharmacy, general stores, and administrative departments. We interviewed department heads, administrators, and accounting staff to determine how departments maintained financial and/or inventory records. Each department maintained an independent records system. Records ranged from January 2016 to June 2018 and covered an average of 17 months per department. Administrators provided spreadsheets with costs data for personnel salaries and budgets for recurrent trainings relevant to EHS for the most recent year (2018).

#### Step 7: collect costs data

We exported records from each department into Microsoft Excel (Redmond, Washington). We asked staff to qualitatively evaluate records’ accuracy and completeness, and we restricted our exports to time periods where staff evaluated records to be accurate.

All departmental records contained lists of line items for purchases made by UNC Project. Line items were not coded or organized in a way that allowed us to automatically disaggregate EHS expenses, and we therefore coded all records by hand. We extracted unique item descriptions from each set of records using *R* statistical software version 3.5.0 (Vienna, Austria). We reviewed and coded all unique line items (*n* = 9290) to identify any expenses relevant to an EHS. Within each EHS, we subsequently coded these relevant expenses into the cost categories described in Table 1. All coding was done independently by two reviewers, and we resolved disagreements by discussion. Codes were then merged into the original data files. Budgets for recurrent trainings and personnel costs were already EHS-specific and required no additional data cleaning.

#### Step 8: aggregate and evaluate

To assess the accuracy and representativity of cost data, we used the iterative process described by Anderson et al. [5]. In brief, following completion of Step 7, we aggregated data across all records and compared it to our cost frameworks. Where frameworks described expenses that were missing from aggregated data, we discussed with administrators to identify the reasons for missingness and identify additional data sources to retrieve missing information, where available. We iterated Steps 6–8 until aggregated data contained all the expenses described in the frameworks, or we determined that any missing data were unavailable. We assessed completeness in terms of presence/absence of expenses and not relative magnitudes.

#### Step 9: calculate costs

We calculated costs at the network level, aggregated across all facilities. Records tracked central purchasing of goods and services at the network level. Disbursement of resources to individual facilities was not tracked in these records, and we therefore did not estimate costs at the facility level.

We divided costs into capital hardware and software costs to establish EHS and annual operations and maintenance costs. We found no financing costs relevant to EHS and therefore excluded this category from our cost calculations. We also found little information on capital hardware and software costs in records. Thus, we do not present a full summary of these costs in the results below, though the full datasets for each EHS, including capital costs where available, are provided in Additional files 6-13.

Annual operations and maintenance costs comprised the costs categories of capital maintenance, recurrent training, consumables, personnel, direct support, and contracted services. For capital maintenance, consumables, personnel, and contracted services, we calculated the average monthly cost based on the data period and multiplied by 12 to obtain annual costs. We used annual budgets for safety trainings for recurrent training costs. Safety trainings were relevant to four EHS: hygiene, PPE, cleaning, and waste management. While curricula for safety trainings covered all EHS simultaneously, we included the full training costs under each EHS, as each EHS required annual training, and costs would not be substantially reduced by removing irrelevant curricula. Waste management had additional costs associated with consumables and capital maintenance for waste transport vehicles, which we estimated using established methods [39].

Personnel costs were adjusted based on the proportion of effort dedicated to each EHS (e.g., clinic aides spent approximately 35% of working hours per week cleaning, so we allocated 35% of their annual salary). Personnel costs included salaries only, not employment benefits. For direct support, we estimated the proportion of effort for supervisors, procurement officers, and logistics officers involved in EHS provision at 1% for EHS requiring substantial supervision of personnel (cleaning and waste management) and 0.5% for all others.

All costs recorded in Malawi kwacha (MWK) were converted into United States dollars ($) using the exchange rate for January 1 in the year during which costs were incurred (2016: $1 = 615 MWK; 2017: $1 = 715 MWK; 2018: $1 = 725 MWK). All costs were adjusted for inflation to January 2019.

Additional calculation details are in Additional file 3.

#### Step 10: share and apply

We conducted an internal review of data with facility stakeholders. We presented records to administrative and accounting staff and asked them to verify that data were accurate and complete to the best of their knowledge and that missing data were not readily available in records systems.

## Results

### Cost frameworks - resource inputs for EHS delivery

Table 2 presents an excerpt of the framework for hygiene, demonstrating how we organized inputs for each essential output into cost categories. Columns for observed and missing expenses in Table 3 overview the resource inputs for each EHS. Full frameworks for each EHS are in Additional file 4.

**Table 2:** Except of the framework of essential outputs and inputs for hand hygiene. The full framework is provided in Additional file 4

Essential outputs	Capital hardware	Capital software	Capital maintenance	Recurrent training	Consumables	Personnel	Direct support	Financing
Hand hygiene at the point of care	Sink or other handwashing facility Dispensers for alcohol-based hand rub Disposal bins for hand drying materials	Site assessment, engineering/architectural design, and planning for sinks Orientation for hand rub formulation and restocking	Sink maintenance and repairs Hand rub dispenser maintenance and repair	Training on proper handwashing technique and handwashing promotion	Soap Alcohol-based hand rub Hand drying materials Utility costs for sink operation	Staff for restocking hand rub, soap, hand drying materials Staff for disposing and/or recycling hand drying materials	Monitoring and inspections of hand hygiene compliance	Interest on loans for sink installation

**Table 3:** Annual costs of environmental health service provision within a network of private healthcare facilities in Lilongwe, Malawi. Due to the high proportion of expected but missing expenses, total annual costs are substantial underestimates of true costs. Costs are reported in 2019 United States dollars ($) and have been rounded to the nearest dollar. Total annual cost and breakdown by cost category may not match precisely due to rounding

Service	Total annual cost	Breakdown by cost category	Expenses observed	Expenses missing or substantially incomplete
Water	$32,878	Capital maintenance: $548 Consumables: $31,272 Direct support: $1057	Consumables for water utility bills, bottled water, disposable cups Direct support from supervisory, logistics, and procurement staff	Maintenance and repair of water main connection, distribution pipes, access points at point of care and for drinking, emergency storage^a^ Recurrent training for water safety and testing Consumables for water testing and treatment Personnel costs for water testing and treatment, monitoring and filling of emergency water storage
Sanitation	$7707	Capital maintenance: $849 Consumables: $5800 Direct support: $1057	Consumables for anal cleansing materials Direct support from supervisory, logistics, and procurement staff	Maintenance and repair of menstrual hygiene facilities Maintenance and repair of toilets, septic tanks, sewer/septic tank connection pipes^a^ Consumables for utility costs for toilet operation and menstrual hygiene management facility; soap, drying materials, and menstrual products (e.g., sanitary pads) for menstrual hygiene management
Hygiene	$23,989	Recurrent training: $1821 Consumables: $21,110 Direct support: $1057	Annual safety training^b^ Consumables for soap and hand drying materials Direct support from supervisory, logistics, and procurement staff	Maintenance of sinks and hand rub dispensers Recurrent training for hand hygiene promotion materials at defecation sites Consumables for alcohol-based hand rub and utility costs for sink operation
Personal protective equipment (PPE)	$26,310	Recurrent training: $1821 Consumables: $23,432 Direct support: $1057	Annual safety training^b^ Consumables for disposable PPE (gloves, masks, aprons, gowns, face shields) Direct support from supervisory, logistics, and procurement staff	Personnel costs for decontamination, sterilization, and restocking of reusable PPE included under waste management
Waste management	$46,752	Capital maintenance: $6735 Recurrent training: $1821 Consumables: $13,621 Personnel: $22,273 Direct support: $2114 Contracted activities: $189	Annual safety training^b^ Consumables for waste collection, segregation, and transportation (disposable waste containers, waste labeling materials, vehicle fuel) and disposable PPE Capital maintenance of waste transportation vehicles Direct support from supervisory, logistics, and procurement staff Contracted services for waste treatment and final disposal	Capital maintenance for collection, storage, transportation trolleys/carts, and treatment equipment^a^ Consumables for utilities for autoclave operation Consumables for disposable PPE for waste handling Direct support for immunizations for waste handlers, waste weighing scale Contracted services for fuel contributions for incineration operation
Cleaning	$69,372	Recurrent training: $1821 Consumables: $17,391 Personnel: $46,968 Direct support: $2114 Contracted services: $1078	Consumables for cleaning chemicals (antiseptics, disinfectants, soaps, and detergents) and supplies (disposable cloths) Personnel for all cleaning non-contracted activities Annual safety training^b^ Direct support from supervisory, logistics, and procurement staff Contracted services for carpet cleaning in office areas	Capital maintenance of sluice room and sterilization equipment Consumables for disposable PPE (aprons, gloves, face shields, masks, gowns) Direct support for immunizations for cleaners
Laundry	$8482	Direct support: $1057 Contracted services: $7425	Direct support from supervisory, logistics, and procurement staff Contracted services for laundry of laboratory coats	None
Vector control	$4937	Consumables: $473 Direct support: $1057 Contracted services: $3407	Consumables for insecticides Direct support from supervisory, logistics, and procurement staff Contracted services for fumigation for insects and rodents	None

^a^ Indicates that some expenses for the described item were included but judged to be substantially incomplete

^b^ Total cost of annual safety trainings was $1821 for all safety trainings conducted within the facility network. Full cost of safety trainings are included under each relevant EHS (hygiene, PPE, waste management, cleaning)

Through identifying essential outputs and inputs for EHS delivery in framework development, we found that EHS divided into two categories—“capital-heavy” versus “personnel-heavy”—based on the predominant type of resources required for service provision. Capital-heavy EHS required substantial resources for upfront capital hardware, capital software, and ongoing capital maintenance but fewer resources for personnel and consumables. They comprised water, sanitation, hand hygiene, waste management, and laundry, which required infrastructure installation with substantial costs (e.g., water and sewer mains, sinks, incinerators, and washing machines). Personnel-heavy EHS were those with minimal resources required upfront to establish services, but higher demand for personnel in routine operations. They comprised PPE, cleaning, and vector control.

Resource needs for consumables were high for personnel-heavy EHS (e.g., need for chemicals for surface disinfection), though waste management and hand hygiene also required substantial consumable resource inputs. Resource needs for recurrent training were comparable across EHS. Direct support outputs were most commonly safety planning, monitoring, and inspections, for which the primary expenses are staff time, though some direct support required purchase of physical goods and services (e.g., waste weighing scales, immunizations for waste handlers).

### Costs of EHS delivery

We provide summary measures of recorded expenses and identify missing data in Table 3. Additional files 6-13 breakdown the line items for each EHS by cost category.

#### Initial costs to establish services

Records contained little information on the capital hardware and software costs associated with the estabilishment of EHS. Often, capital hardware costs were for infrastructure purchased and installed as a part of facility construction (e.g., water distribution pipes, indoor flush toilets), and construction-related costs were not included in electronic records that we obtained for this study. Relevant capital software costs (e.g., site assessment, engineering design, procurement, and licensing) were similarly incurred at the time of construction and not in electronic records.

For the capital hardware and software costs of equipment purchased after facility construction, records captured few relevant costs. Several low-cost items for PPE, cleaning, and waste management were purchased frequently throughout the timespan of available records (PPE: $1796 for reusable gloves, aprons, coveralls, and protective boots; cleaning: $2546 for brooms, brushes, mops, and buckets; waste management: $1681 for waste receptacles) but were not representative of overall capital hardware costs.

For most capital hardware items with higher expected expenses (e.g., autoclaves or incinerators) available records typically did not contain relevant expenses, likely because items were purchased before records systems were established and their lifespan exceeded the duration of records. We found some recorded examples of capital hardware costs, such as $116 for the purchase of a waste trolley. However, as many expected capital hardware costs were not captured, we do not provide summary measures here. Evidence for the few examples we did find documented are included in Additional files 6-13.

#### Annual operations and maintenance expenses

Summaries of the total annual operations and maintenance costs for each EHS are presented in Table 3. Annual network-wide costs for all EHS totaled $220,427. For all EHS, observed costs are likely substantial underestimates of the full financial costs of EHS provision, in part due to missing information. For each EHS we summarize the expenses represented in overall costs and expenses that were expected but missing.

Annual operations and maintenance costs were highest for cleaning and waste management, though they had the fewest missing expenses. For both, personnel costs were a substantial proportion of overall expenses. Subsequent sections provide a breakdown of record-keeping practices and available data by cost category.

Most capital maintenance costs were missing from records. We relied on line-item descriptions to identify and extract capital maintenance costs, and these descriptions were often insufficient to identify the type of maintenance performed or determine its relevance to EHS. For example, we found line items for unspecified building maintenance and repairs, but rarely was the work type or repair target described. Where the specific nature of the maintenance was described and relevant to EHS (e.g., “repair of blockage to toilets,” categorized as sanitation), we included these line items as capital maintenance expenses. Due to the high frequency of unspecified maintenance and repair expenses, we judge these capital maintenance costs to be substantial underestimates.

Recurrent training comprised an annual “safety” training, covering topics related to safe handling of hazardous materials (e.g., infectious and sharps waste), laboratory and clinical specimens, and proper use of PPE. Training costs included facilitator fees, allowances for attendees, and food. Training curricula were relevant to hygiene, PPE, waste management, and cleaning, and we included the full training costs under each of these EHS. We found no record of recurrent trainings for other EHS, although we expected recurrent training for all EHS. Total annual costs of safety training were $1821 for three training sessions, one each for clinical and pharmacy staff, laboratory staff, and clinical support staff and waste handlers.

Consumables contributed the most to overall annual operations and maintenance expenses for all EHS except waste management and cleaning; and consumables were the expenses most consistently recorded in electronic records. Consumables costs were highest for water ($31,272) and PPE ($23,432). Water consumables were predominantly water utility bills ($27,540), while PPE consumables were more diverse. Consumable costs for vector control were several orders of magnitude lower than other EHS ($473), and vector control costs were primarily for contracted services.

We identified personnel costs for cleaning and waste management totaling $46,968 and $22,273 annually, respectively. Clinic aides were primarily responsible for cleaning and waste management activities, but nurses, laboratory and pharmacy technicians performing supervisory and specialized tasks (e.g., cleaning of specialized laboratory and clinical equipment) contributed a higher percentage of costs due to the high number of nurses employed and their higher salaries. Interviews suggested that the proportional effort that staff dedicated to other EHS was negligible and was therefore not included (water, sanitation, and hygiene), or personnel costs were included in contracted services and could not be disaggregated (laundry and vector control).

Direct support costs were estimated as $1057 each for water, sanitation, hygiene, PPE, and vector control, and $2114 for cleaning and waste management. These costs represent the proportional effort of supervisory, procurement, and logistics staff. Costs of goods and external services required for direct support (e.g., water quality test kits, waste transportation vehicle insurance) were not available in records and are not included in cost estimates.

## Discussion

We applied a model designed specifically for costing EHS [5] in a network of facilities that provide clinical care within the context of research and training in urban Malawi. We extended this model by developing frameworks of the essential outputs and inputs for each EHS through review of international guidelines for EHS in HCFs. We considered the inputs in each framework to represent the line item expenses essential for EHS delivery. We then applied these frameworks to assess the completeness of the costs information recorded in HMIS of the HCF network by assessing whether these essential expenses were present or absent from records.

For all EHS, we found that a substantial number of essential expenses were missing from records—particularly capital hardware, capital software, and capital maintenance costs—indicating that the cost estimates reported here are substantial underestimates of the full financial costs of EHS delivery within the HCF network.

We attribute missing information in the electronic records to several factors. Capital hardware and software costs incurred at the time of facility construction were not documented in the electronic records obtained for this study. Contractors maintained detailed records of construction-related costs at the time of construction, which contained EHS-related expenses, but only summaries were retained by UNC Project. Any item or service purchased by UNC Project from UNC-Chapel Hill directly is not recorded in systems from which we obtained records. In particular, large equipment for capital hardware is typically purchased directly through UNC-Chapel Hill purchasing services, and these transactions are not recorded by UNC Project. Obtaining records from outside the UNC Project system was beyond the scope of this study. However, future studies could document these costs by prospectively documenting relevant costs for new construction or major rehabilitations, contacting contractors to obtain additional records or estimates, or examining audited financial statements as potential data sources. In calculating capital costs, future studies will also need to take additional steps to properly account for depreciation of hardware.

Records only covered an average of 17 months of data, which is likely insufficient to capture infrequent expenses. Capturing lifecycle costs of EHS remains a challenge, as the lifespan of EHS infrastructure can be several decades, making prospective data collection difficult. Electronic records systems for retrospective data collection are uncommon in Malawi and other LMICs [40], and lack of workers trained to use these systems remains a barrier [41]. Efforts to strengthen HMIS will likely be necessary for widespread data collection in LMICs. Further research across EHS in different phases of the lifecycle with shorter time frames could be used as an alternative.

We attribute missing information on the costs associated with operations and maintenance in part to inconsistent labeling and disaggregation of EHS expenses and completeness of records. For example, capital maintenance costs were often missing because records did not contain sufficient detail to determine the type of maintenance performed and attribute it to a specific EHS. Another plausible explanation for missing expenses is inadequate service delivery (i.e., necessary resource inputs were not being purchased and provided). Evidence from public facilities in Malawi indicates that shortages of PPE, soap for handwashing, and other EHS are common [42–44]. Similarly, practices such as open dumping of waste are common in Malawi and other LMICS [9] and will have substantially lower costs compared to appropriate treatment and disposal. However, in our contextual assessment (Step 4) we found that the essential outputs that we identified during framework development were typically present at UNC Project. As such, we find this explanation unlikely to be a substantial contributor to underestimates in the context of UNC Project.

We identified personnel costs only for cleaning and waste management. Laundry and vector control would have likely incurred substantial personnel costs if provided in-house, but they were paid as a single fee for contracted services that covered all costs categories. In our qualitative assessment from Step 4, we determined that the personnel costs associated with other EHS were negligible. However, frequent tasks individually requiring little time (e.g., restocking drinking water dispensers or time spent washing hands) contribute to costs and in large facilities may sum to substantial amounts. Time and motion studies, activity diaries, or other observational techniques could be used to identify these costs [45].

We classified some expenses as capital hardware but observed frequent repurchasing, notably for cleaning tools (e.g., mops, brooms), reusable PPE, and waste bins. These items fit our definition of capital hardware in that they “are not consumed during normal service operation.” However, frequent repurchasing suggests they have a short lifecycle and/or are disposed rather than repaired. Given frequent repurchasing, these items may alternatively be considered consumable costs. When considered consumable products, these items respectively contribute $1197, $1121, and $1698 to PPE, waste management, and cleaning operations and maintenance costs annually (see Additional files 9-11).

For contracted services, we did not attempt to contact contractors to assess individual line items. Facilities are likely to contract services when doing so is cost-effective in comparison to in-house provision. In these cases, contractor fees may underestimate the costs of providing the same service in-house, as economies of scale likely apply to contracted services. In some cases, subsidies for services contracted to the government may underestimate the costs of service provision. This is likely true with waste management at UNC Project, which was contracted to the government hospital at a substantially lower cost than found in other settings (see, e.g., [46, 47]). For water and sanitation, utilities in LMICs charge tariffs substantially below full cost recovery [48].

From our framework development, we found that EHS fell into two type of expense profiles: capital-heavy versus personnel-heavy. We found cleaning, vector control, and PPE were personnel-heavy. However, this distinction will be sensitive to several factors, including the modality of service provision and relative prices of labor versus goods. For example, we found hand hygiene to be a capital-heavy EHS due to infrastructure inputs for sinks with piped water. Hand hygiene provided through lower-cost infrastructure that is not connected to a piped network may shift the majority of costs to other categories.

Whether EHS are capital- or personnel-heavy has implications for investment in EHS improvements. Personnel-heavy EHS will have lower upfront financial barriers to establish or conduct major infrastructure rehabilitations or improvements, and may therefore be more feasible in settings where funds for substantial capital investment are lacking. Research on lifecycle costs, as well as cost-effectiveness of various EHS, is lacking but would inform investment decisions [5]

### Limitations

This study captures only the costs that were recorded in electronic records systems. A majority of capital hardware and capital software expenses were missing from these records, as well as a substantial number of expenses from other costs categories. A bottom-up costing approach, which identifies the unit costs and quantities of each resource input, would allow for more granular detail regarding specific expenditures [49], but we found that bottom-up costing was infeasible given poor recall and the large number of products used in EHS provision.

As UNC Project provides healthcare in the context of clinical research trials, the profile of expenses in non-research focused HCFs may differ. UNC Project employs a high number of administrative and research personnel that do not interact with patients. These personnel and their associated building spaces contribute to EHS costs differently than clinical staff and spaces (e.g., through production of exclusively general, non-hazardous waste). Public HCFs in Malawi would be more generalizable to HCFs in other LMICs. However, during pilot testing we found that recall and paper-based records in public facilities were insufficient to support costing. LMICs where public facilities have stronger electronic HMIS are likely more feasible settings for future costing research.

We were unable to account for how resources were used within UNC Project after purchase at the network level. UNC Project facilities are separate buildings or designated rooms solely for UNC Project use within government facilities. However, some UNC Project staff did work in the wards of government-run facilities and collaborate with public HCF employees. While UNC Project was not responsible for ensuring adequate EHS outside of designated rooms in government facilities, our interviews indicated that resources from UNC Project were sometimes diverted to compensate for inadequate conditions in government facilities. For example, although the government was responsible for ensuring functional toilets in buildings where UNC Project had designated rooms, UNC Project reported paying for repairs when the government was delayed or unable to ensure functionality. We were unable to determine the value of goods diverted out-of-network but recognize that this will result in overestimates of the true costs of achieving adequate EHS in UNC Project facilities.

We categorized expenses under the EHS where we judged them to be most used, based on our frameworks. For example, we classified all recorded PPE expenses as PPE at the point of care, although we know some proportion of PPE were used for cleaning, waste management, and potentially other applications. We assumed that products were used as marketed. For example, we assumed that surface cleaning soaps were used for surface cleaning and hand soaps were used for handwashing, although in some cases these products may be used interchangeably. This may result in minor misclassification of items and error in the costs per EHS but should not change the combined total for all EHS.

## Conclusions

We applied a process model designed specifically for costing EHS in HCFs [5], and we extended this model by developing frameworks of essential the outputs and inputs for EHS to support costing. We developed and applied an interview guide to assess facility context and EHS quantity and quality. This is the first study to use a formal model and frameworks to guide costing of EHS in HCFs in LMICs, and we encourage future research to apply these methods and tools in other settings.

We attempted to estimate the costs to establish, operate, and maintain eight EHS, using available electronic records. However, due to record-keeping practices and short timeframes of available records, our estimates for certain cost categories are likely substantial underestimates. Data collection through other means, for example bottom-up costing using surveys and observations, may yield more complete estimates. However, bottom-up costing can compensate for limitations of records but can be challenging due to high staff turnover and poor recall, and is time- and resource-intensive. Electronic HMIS have the potential to facilitate more efficient data collection, but our research suggests a need to better code and disaggregate EHS expenses to fully leverage these systems.

We used our costing frameworks primarily to assess completeness of records. We assessed only presence/absence of expenses, but our frameworks could be adapted to assess magnitude. Such an adaptation would require additional research on the correlation between variables such as facility size, patient volume, and type of healthcare services offered, which influence the quantity and quality of EHS needed but in ways that are poorly understood. Given low priority often assigned to EHS in low-resource settings [50, 51], documenting resource inputs can be used to identify instances where necessary resources are not consistently available and to estimate the additional expenses that would be needed to achieve adequate EHS conditions. We identified general categories of resources inputs rather than specific products (e.g., surface disinfectants for cleaning, rather than specific products or brands) so that these frameworks may be applied for costing in other settings. However, some frameworks may require adaptation in HCFs using other modalities of EHS provision, such as on-premises boreholes instead of utility piped water. While we have not specifically designed our frameworks for this purpose, future research could adapt these or develop similar frameworks to monitor safety and adequacy of service delivery.

## Supplementary Information

**Additional file 1.** EHS & facility building descriptions

**Additional file 2.** Interview guide and participant descriptions

**Additional file 3.** Additional costing methods

**Additional file 4.** EHS costing frameworks

**Additional file 5.** CHEERS checklist

**Additional file 6.** Disaggregated costs for water

**Additional file 7.** Disaggregated costs for sanitation

**Additional file 8.** Disaggregated costs for hygiene

**Additional file 9.** Disaggregated costs for PPE

**Additional file 10.** Disaggregated costs for waste management

**Additional file 11.** Disaggregated costs for cleaning

**Additional file 12.** Disaggregated costs for laundry

**Additional file 13.** Disaggregated costs for vector control

## Data Availability

The datasets supporting the conclusions of this article are included within the article and its additional files.

## Abbreviations

EHS: Environmental health services
HAI: Healthcare-acquired infection
HCFs: Healthcare facilities
HMIS: Health information management systems
JMP: Joint Monitoring Program
LMICs: Low- and middle-income countries
PPE: Personal protective equipment
SDGs: Sustainable Development Goals
UNC: University of North Carolina
WHO: World Health Organization
