# Supplementary material for: Development and application of tools to cost the delivery of environmental health services in healthcare facilities: a financial analysis in urban Malawi
Source: BMC Health Serv Res. 2021 Apr 13;21:329. doi: 10.1186/s12913-021-06325-3 (PMC8042714; doi:10.1186/s12913-021-06325-3)
Supplement: Supplementary file 1 — Additional file 1. EHS & facility building descriptions [file 12913_2021_6325_MOESM1_ESM.docx]

# UNC Project buildings

## Clinical care buildings

- Area 18 George Joak Center: 9 exam rooms, 1 clinicians’ office, 1 data room, 1 laboratory, 2 staff rooms, and a kitchen
- Area 25: operates out of designated and separately metered space in a government facility, contains 2 UNC clinic rooms
- Bwaila STI clinic: 8 exam rooms, 4 non-clinical survey rooms, records office
- Bwaila shipping container: 3 exam rooms
- Bwaila Promote Building: 10 exam rooms
- Bwaila S4 Building: 4 exam rooms
- Tidziwe Center: pharmacy, laboratory, 24 office rooms, 2 conference rooms, 1 lecture hall, and 26 exam rooms

## Non-clinical buildings

- UNC Storage warehouse: document and supply storage space, no exam rooms, electricity but no water connection
- Tidziwe Annex: three-story research facility with ground-floor laboratory, freezer, and data storage areas, and first- and second-floor office and conference space (approximately 370 m^2^ per floor)

UNC Project staff additionally work in the wards at Kamuzu Central Hospital (KCH), though UNC Project is not officially responsible for maintaining EHS in these facilities. Kamuzu Central Hospital dedicates 6 rooms in pediatrics and 2 rooms in oncology specifically for UNC Project care and research activities, though UNC Project staff also work in other wards without dedicated rooms.

# UNC Project staff

The following staff providing were employed in January, 2018:

Clinical, laboratory, and pharmacy

- Specialists, medical officers, clinical officers: 29
- Research nurses: 68
- Pharmacy staff: 8
- Laboratory staff: 30
- Clinic aides: 14
- HIV testing and counseling: 12

Other

- Data management: 75
- Regulatory staff: 2
- Information and communications technology: 3
- Accounts: 11
- Security: 51
- Administration: 12
- Transportation: 18
- Gardeners: 4
- Guesthouse keepers
- Community educators: 8
- Tracers: 3
- Management: 5

Total permanent employees: 355

# Clinical services profile

The following medical research activities were recorded in 2018

- 50 active research studies
- 19,913 study-related visits

The following clinical care activities were recorded in 2018:

- 25,849 patient care hours
- 7,268 pathology reports
- 42,228 patients encountered

The following types of care were provided at UNC Project

- Pediatrics (6,410 patients encountered)
- Infectious diseases/sexually transmitted infections (28,800 patients encountered)
- Obstetrics and gynecology (2,702 patients encountered)
- Oncology (4,316 patients encountered)
- Laboratory (no patients encountered, 5,388 service hours)

| **Facility** | **Clinic type** | **number clinical staff** | **patient visits per week** |
| --- | --- | --- | --- |
| Bwaila | STI | 8 | 250 |
| KCH | sickle cell | 5 | 60 |
| KCH | cancer | 6 | 100 |
| Tidziwe | HIV research | 12 | 80 |
| Bwaila | HIV research | 10 | 100 |
| Area 18 | HIV research | 5 | 75 |
| KCH | Pediatrics | 6 | 50 |
| Bwaila | Pediatrics | 4 | 20 |
| KCH | OB/GYN | 3 | 50 |

# Water

Lilongwe water Board water is used for all purposes except drinking, and water is piped into the facilities. Drinking water is provided through dispensers and refillable 20L bottles purchased from local water companies or grocery stores. Project makes water utility payments at all buildings described above, except Kamuzu Central Hospital.

Water storage towers with tanks are available at Tidziwe, Area 25 and Area 18. Bwaila does not have a storage tank and relies on clinic staff to fill buckets when water is anticipated to run short.

When maintenance is needed, clinic staff report the problem to Administration, who is responsible for hiring contracting maintenance to repair (for repairs to internal pipe distribution systems) or reporting the problem to the Lilongwe Water Board (for repairs to water mains or other external infrastructure).

For most applications, water quality is not tested. No supplemental treatment is done to the piped water supply, as it is assumed to be safely treated by the water board. When distilled water is needed for laboratory procedures, the laboratory has infrastructure in-house for distillation. The laboratory also supplies sterile water for specific medical procedures when necessary. Costs of distillation and water treatment for laboratory procedures and kept by a separate records system for the laboratory. Water bills to the water board are paid by the Administration department.

# Sanitation

Sanitation at Tidziwe uses indoor flush toilets for both patients and staff. Bwaila, Area 18, and Area 25 have flush toilets for staff and pit latrines for patients. Maintenance of toilets is arranged by Administration. Clinic aides are responsible for cleaning toilets, and when they find that toilets are broken, they report to Administration, who arranges repairs.

Sewage is stored in septic tanks for Tidziwe and Area 18. At Bwaila, UNC uses space in a larger government facility, and the government should be responsible for maintaining all infrastructure. However, often repairs are not timely when funded by the government, and UNC Project will fund repairs as needed to ensure that services are provided.

# Waste management

## Collection, Segregation, Storage, Transportation, and Treatment procedures

Waste is sorted in the clinic into hazardous and general waste. Sharps waste is placed in sharps containers, which are added to the hazardous waste when approximately three-quarters full. Infectious waste is sorted into hazardous waste.

Hazardous waste from Tidziwe, Bwaila, and Area 18 are combined. Hazardous waste is collected from clinic rooms and laboratory and loaded into a waste trolley. Trolley contents are transported to a storage room with an autoclave at Tidziwe. Waste is stored in large bins in the storage room and sterilized in the autoclave weekly. Bwaila and Area 18 bring biohazard waste to Tidziwe on each day the clinics are open (Monday – Friday, closed weekends and public holidays).

Following sterilization, waste is loaded onto a truck. Waste is held on the trunk and then driven to a government incinerator for incineration and disposal. Biohazard waste is transported to incinerator and burned monthly (10,000 MKW per month)

UNC Project contributes fuel to offset the costs of incinerator operation. The truck is used exclusively for waste transport purposes. The Administration department is responsible for operation and maintenance costs of the autoclave and truck. The government should be responsible for maintenance of the incinerator, but repairs can be slow. If the incinerator is not functioning and the government is slow on repairs, UNC Project will finance repairs to get the incinerator working again. Administration is responsible for arranging costs regarding the incinerator.

Area 25 gives their waste to the government partners sharing the facility, and the government is responsible for waste management.

At all facilities, general waste is taken to an open burning pit located on-site and burned daily. Oversized general waste (e.g. office furniture) is reported to administration, and administration is responsible for arranging disposal of large items.

Pharmaceutical waste is quarantined when drugs expire or otherwise need to be disposed. Quarantined waste is brought to Kamuzu Central Hospital when there is sufficient volume for disposal, on average quarterly. UNC Project pays KCH 10,000 MKW per disposal use, or 40,000 MKW per year. Pharmacy writes a requisition to Administration, who provides funding. Kamuzu Central does not generate receipts, so no formal record of payment exists.

## Personnel

Nurses decontaminate if necessary and segregate waste into biohazard, general waste, and sharps waste. Clinic aides subsequently collect the biohazard waste from clinic rooms and transport it to the sterilization room. Laboratory cleaners collect hazardous waste from the laboratory. One clinic aide is responsible for running the sterilization and requesting money from Administration to pay for costs of incinerator. Clinic aides collect general waste and transport it to the pit on-site for burning. Normally the fire is always burning, so waste is just thrown on and left to burn. If the fire is not burning, a clinic aide must supervise rekindling the fire so that waste can be added and burned.

UNC hires drivers to transport waste to the government incinerator. When drivers are hired for waste transport, they must receive training for infection prevention for safe waste management. Clinic aides also receive training for safe waste management. Infection prevention safety training is conducted yearly. Training is organized as a joint venture by clinic management and the Laboratory department, and copies of budget are kept on record with the Laboratory department.

Supplies for biohazard waste management are order through Laboratory department, and general waste management is ordered through the Stores department. Occasionally somethings are ordered through pharmacy.

Staff involved in waste management comprise:

| Staff | Role | % effort of all duties |
| --- | --- | --- |
| Nurses | Waste collection and segregation at point of care, Supervision of waste management | 2% |
| Laboratory and pharmacy technicians | Waste collection and segregation in laboratory and pharmacy  Supervision of waste management | 2% |
| Clinic aides | Waste transportation within-facilities  Storage and autoclave processing | 15% |
| Laboratory cleaners | Waste collection in the laboratory  Waste transportation within-facilities | 30% |
| Drivers | Transportation of waste between facilities | 2,311 hours of driver time per year |

# Cleaning

## Procedures

Decontamination of patient surfaces is done by nurses when spoiled, or at a minimum daily if never spoiled. Cleaning logs are kept in each room. Clinic aides are responsible for initial cleaning of medical equipment. Clinic aides bring equipment to the sluice room for initial cleaning. After cleaning in the sluice room, equipment is packaged and sent to the laboratory for sterilization. Once sterilized in the laboratory, clinic aides collect the equipment and return it to the clinic. Nurses will clean some specialized medical equipment in clinic rooms. General cleaning of non-clinical spaces is done by clinic aides.

Cleaning of specialized equipment within the pharmacy is done exclusively by the pharmacy technicians. General cleaning of the pharmacy is done by clinic aides. The laboratory has their own dedicated cleaners for laboratory equipment.

Cleaning supplies for the clinic are purchased through laboratory and pharmacy. General clinic supplies are purchased through stores. General cleaning of the pharmacy is done by clinic aides, supervised by the head nurse. Purchase of specialized medical cleaning supplies (e.g. disinfectants) is done through the pharmacy. General cleaning supplies are purchased through Administration, under the supervision of the head nurse.

## Personnel

Staff involved in cleaning comprise:

| Staff | Role | % effort of all duties |
| --- | --- | --- |
| Nurses | Cleaning specialized equipment  Supervision of cleaning | 5% |
| Laboratory and pharmacy technicians | Cleaning specialized equipment  Autoclave operation (lab techs only)  Supervision of cleaning | 5% |
| Clinic aides | General cleaning of clinical, pharmacy, and non-clinical spaces  Initial processing of equipment for autoclaving | 35% |
| Laboratory cleaners | General cleaning of laboratory spaces  Cleaning of glassware and laboratory supplies  Autoclave operation | 70% |

# Hygiene

Handwashing sinks are available at points of care. Hand drying in clinic is done with paper towels, which are disposed through general waste. Alcohol hand rub stations are also available throughout the clinic, and purchase of supplies for these is done through laboratory. Hand drying at toilets is a mixture of electronic air hand dryers or no facilities. Maintenance of handwashing facilities is supervised through Administration.

# Personal protective equipment

PPE is worn by all staff except for Administration when cleaning or handling waste. The specific type and procurement procedures for PPE vary by department. Nurses, pharmacy staff, and clinic aides purchase PPE through Administration. Laboratory staff and clinicians purchase PPE through Laboratory department. Driver PPE is purchased through the transportation officer via General Stores.

When PPE is cleaned and reused, clinic aides are responsible for cleaning. Some are cleaned in-house, while others are contracted to external laundry services.

# Laundry

Laundry is generally contracted to an external facility, though some small items are washed by hand by clinic aides. Administration manages contracting for laundry. Laboratory generates most of the laundry.

# Vector control

Fumigation services for insect vectors are contracted through Administration. All vector control services are contracted. Occasionally bed nets are purchased through UNC project, but these are given to households for studies run by UNC Project that are operated in the community.

# Safety trainings

Safety trainings are run by laboratory and the chief nursing officer. Budgets for trainings are available in the laboratory department. Three trainings are run annually, one each for clinical and pharmacy staff, laboratory staff, and clinic aides and drivers. Topics vary by training but include information on standard precautions at the point of care, safe handling of clinical and laboratory specimens, safe handling of waste, and proper use of PPE. Safety trainings are relevant to cleaning, waste management, hygiene, and PPE.

# Maintenance expenses

All maintenance work is contracted. No plumber, electricians, maintenance workers, etc. are employed by UNC Project. Administration maintains a list of contractors who are called when repairs are needed—typically it’s the same man who comes to do maintenance. This is true for clinic spaces, pharmacy, and laboratory. Groundskeepers will sometimes be engaged for minor repairs (e.g., unclogging drains), when it is within their capacity.

# Note on spending and budget disbursement

UNC Project will subsidize government facilities where UNC is collaborating if the government is unable to provide the necessary equipment. For example, UNC nurses collaborating in government facilities will have PPE provided through UNC, but government nurses employed on the same project may lack PPE. When this happens, UNC Project will also provide PPE for nurses employed by the government who are collaborating on the project. Trainings for UNC staff for infection prevention will include government staff who are also working on the project, but the government does not contribute to the costs of training. UNC Project also subsidizes infrastructure at government facilities. For example, when the sewage system at Bwaila breaks down and government repair is slow, UNC Project will subsidize repairs of the system because participants in UNC studies need those facilities and UNC does not want to lose study participants because facilities that are supposed to be maintained by the government are not functioning.
