# Supplementary material for: Development and application of tools to cost the delivery of environmental health services in healthcare facilities: a financial analysis in urban Malawi
Source: BMC Health Serv Res. 2021 Apr 13;21:329. doi: 10.1186/s12913-021-06325-3 (PMC8042714; doi:10.1186/s12913-021-06325-3)
Supplement: Supplementary file 2 — Additional file 2. Interview guide and participant descriptions [file 12913_2021_6325_MOESM2_ESM.docx]

Participant list

Table 1. Participants interviewed during data collection. Participants are listed in the order interviewed, although some participants were interviewed multiple times. Job descriptions have been generalized to protect interviewee confidentiality.

| **Job description** | **Department** | **Purpose of interview(s)** | **Data provided** |
| --- | --- | --- | --- |
| Senior facility administrator | Administration | Access permissions for records  Identify relevant staff for follow-up interviews | [none] |
| Senior clinical supervisor | Administration | Assess EHS provision at UNC Project and resources uses  Assess personnel involved in EHS provision | EHS quantity and quality assessment  Staff percent-effort dedicated to EHS |
| Accounts supervisor | Accounts | Access permissions for records  Overview of all facility records systems  Identify relevant staff for follow-up interviews  Review all records and assess data completeness | [none] |
| Procurement officer | Stores | Determine records systems for General Stores department  Provide General stores costs data  Assess data completeness | General Stores records |
| Electronic records information technology consultant | Consultant | Troubleshooting access challenges for General Stores records | Unit costs and other missing data from General Stores records |
| Pharmacy staff member | Pharmacy | Determine records systems for Pharmacy department  Provide Pharmacy costs data  Assess data completeness | Pharmacy records |
| Laboratory officer | Laboratory | Determine records systems for Pharmacy department  Provide Pharmacy costs data  Assess data completeness | Laboratory inventory lists  Unit prices for laboratory supplies |
| Laboratory management team member | Laboratory | Provide recurrent trainings budgets  Provide unit costs for select laboratory supplies  Assess data completeness | Recurrent trainings budgets  Unit prices for laboratory supplies |
| Senior human resources officer | Administration | Access permissions for records  Provide data on contract fees paid for waste management | [none] |
| Accounts supervisor | Accounting | Access permissions for records  Overview of records systems, funding streams, tax payments  Identify relevant staff for follow-up interviews  Assess data completeness | [none] |
| Accountant | Accounting | Provided Administration data | Administration records |
| Senior facility administrator | Administration | Access permissions for records  Provide human resources salary spreadsheets | Staff salaries |

Interview guide for EHS assessment

# Objectives

- Document resources required to deliver EHS
- Describe EHS quality

# Water supply

### How is water provided at this facility?

- 1. What is the main source of water? Describe the source.
     1. *Is this source used for drinking water? If no, what is the drinking water source?*
     2. *Is this source used when water is needed for medical procedures? If no, what is?*
     3. *Is this source used for other non-medical purposes, such as cleaning or laundry? If no, what is?*
  2. Does this facility use any other water source?
     1. *If yes, what source is used?*
     2. *How is water from this other source used?*

### How is water accessed and distributed within the facility?

#### How is water accessed by medical providers during care delivery?

- - 1. *What infrastructure is used for distribution within the facility?*
  1. How is water accessed for cleaning or other activities not directly related to patient care?
     1. *Is this method of access different from how medical providers access water? If yes, how?*
  2. How is drinking water accessed by patients or care givers at the facility?
     1. *What infrastructure is provided for patients to access drinking water?*

### How does this facility store water?

- 1. What infrastructure is used for emergency storage?
  2. What other storage infrastructure is used?

### What maintenance and repairs are needed for the water supply system?

#### Who does the maintenance?

### What other activities need to be done to supply water at this facility?

### Who does the water quality testing?

### Who else is involved in ensuring that there is water supply available?

# Sanitation

### How sanitation is provided for patients at this facility?

- 1. What type(s) of toilets are available for patients?
  2. How can female patients or carers dispose of menstrual products within these facilities?

### How sanitation is provided for staff at this facility?

#### What type(s) of toilets are available for staff?

- 1. How can female staff dispose of menstrual products within these facilities?

### Are there any other sanitation facilities available?

- 1. Who uses these toilets?
  2. What type(s) of toilets are they?
  3. How can female staff dispose of menstrual products within these facilities?

1. **How does this facility manage sewage?**
   1. Are any toilets connected to a sewer or other wastewater drainage system?
      - 1. *[If yes] Describe the sewer/drainage infrastructure.*

### How is other infectious waste water disposed?

- 1. **Does this facility have any other infrastructure to dispose of waste water, either from sanitation facilities or waste water from other sources?**
     1. *[If yes] Can you please describe that infrastructure?*

1. **What maintenance and repairs are needed for the toilets and sewer system?**
   1. Who does the maintenance?

### What other activities need to be done to supply sanitation at this facility?

### Who else is involved in ensuring that sanitation facilities are available?

# Patient and provider hygiene

### Describe the facilities available for handwashing by patients.

#### How do patients dry their hands after washing?

### Describe the facilities available for handwashing by health care providers.

#### How do health care providers dry their hands after washing?

### Are any other facilities available for hand hygiene at the point of care, such as alcohol-hand rub dispensers?

#### [If yes] Describe the facilities available.

### What maintenance is needed for handwashing facilities?

#### Who does the maintenance?

# Waste management

### How is waste collected and stored at the point of care?

### How is sharps waste treated prior to disposal?

#### How is sharps waste collected and transported from the treatment point to be disposed?

### How is infectious waste treated prior to disposal?

#### How is infectious waste collected and transported from the treatment point to be disposed?

### How is waste from the incinerator (or other treatment processes) disposed?

### How is non-infectious, non-sharps general waste disposed?

### How does UNC Project dispose of large items, such as broken office furniture?

### What protective equipment are worn by staff during waste processing?

### What maintenance and repairs are needed for the waste treatment system?

#### Who does the maintenance?

### What other activities need to be done to waste is properly managed?

### Who is responsible for waste disposal activities?

#### Who manages waste at the point of care?

#### Who transports waste from the point of care to storage areas? Who transports waste from the storage area to the treatment point? Who treats waste?

#### Who disposes of waste after treatment?

# Cleaning

### Describe the cleaning process for environmental surfaces in patient care areas.

#### What products are used for cleaning in patient care areas?

#### Who is responsible for cleaning patient care areas?

### Describe the process for sterilizing medical devices

#### What products and equipment are used for sterilization?

#### Who is responsible for sterilization?

### Who is responsible for cleaning non patient care areas?

#### What products and supplies are used in cleaning non-patient areas?

### What protective equipment are worn by staff during cleaning?

### What maintenance and repairs are needed for the sterilization equipment?

#### Who does the maintenance?

### What other activities needed to ensure that the facility is properly cleaned?

### Who else is responsible for cleaning at this facility?

### Who keeps records of purchases of cleaning supplies?

# Personal Protective Equipment

### What protective equipment are used by health care providers?

### What protective equipment are used by cleaners?

### Does anyone else wear protective equipment?

### How is reusable protective equipment cleaned for reuse?

#### Where are reusable PPE processed for cleaning? How is this done?

#### Who does the cleaning?

### Who keeps records of purchases of protective equipment?

# LAUNDRY

### What items need laundering at this facility?

### How is laundry done at this facility?

#### How is laundry handled and processed from the point of use?

#### What infrastructure is used to wash laundry?

### Who is responsible for doing laundry?

### What maintenance is needed for the laundry facilities?

#### Who does the maintenance? Who is responsible for purchasing parts?

# VECTOR CONTROL

### What does this facility do to control insect vectors?

### What does this facility do to control rats, mice, or other rodents?

### Does this facility do any other vector control?

# POWER AND LIGHTING

### How does this facility receive power and electricity?

### Who keeps records of the electricity bill?

### What maintenance is needed for the electrical system and power supply?

#### Who does this maintenance?

### How does this facility get backup power during blackouts?

### What maintenance is needed for the backup power supply?

#### Who does this maintenance?

# HEATING, VENTILATION, AND COOLING

### Is there a ventilation system in this facility?

#### If so, what maintenance does it require?

#### Who maintains it?

### How is this facility heated?

#### What maintenance is needed for the heating system?

#### Who does the maintenance?

### How is this facility cooled?

#### What maintenance is needed for the cooling system?

#### Who does the maintenance?
