# Supplementary material for: Development and application of tools to cost the delivery of environmental health services in healthcare facilities: a financial analysis in urban Malawi
Source: BMC Health Serv Res. 2021 Apr 13;21:329. doi: 10.1186/s12913-021-06325-3 (PMC8042714; doi:10.1186/s12913-021-06325-3)
Supplement: Supplementary file 3 — Additional file 3. Additional costing methods [file 12913_2021_6325_MOESM3_ESM.docx]

# Supporting information file 3

# Cost calculation methods

# Data sources and cleaning

We extracted costs information from the following departments (Table 1)

Table 1. Facility records systems containing costs of environmental health service delivery.

| **Data sources** | **Expense categories** | **Coverage dates*** |
| --- | --- | --- |
| Administrative department accounting software | Capital hardware  Capital maintenance  Consumables | Dec. 2016 – May 2018 |
| General stores department accounting software | Capital hardware  Consumables | Dec. 2016 – May 2018 |
| Pharmacy Department procurement records | Consumables | Oct. 2017 – June 2018 |
| Laboratory Department inventory records | Consumables | Jan. 2016 – Dec. 2017 |
| Budget spreadsheets for annual trainings | Recurrent trainings | Jan. 2018 – Dec. 2018 |
| Human Resources salary spreadsheets | Personnel | Jan. 2018 – Dec. 2018 |
| * Coverage dates are inclusive of all days for the indicated month | | |

## Administrative and General Stores departments

These records contained line item descriptions, dates of purchases, and total transaction costs. Unit prices of goods were identified through visits to in-country and online suppliers. Quantities of goods purchased were calculated as total transaction costs divided by unit cost. Some quantities are not whole numbers due to variation in unit costs. Unit costs and quantities were not applicable to services and tax payments, and this information was left as missing for these line items.

## Pharmacy department

Pharmacy records indicated quantities of goods purchased, unit prices, and dates of purchases. Total transaction costs were calculated as a function of unit price * quantity.

## Laboratory department

Laboratory records indicated quantities of goods purchased and dates of purchases. Unit prices were not recorded, as the system was primarily used for inventory tracking rather than accounting. Unit prices of goods were identified through paper and electronic receipts from laboratory staff and visits to in-country and online suppliers where goods were purchased. Total transaction costs were calculated as a function of unit price * quantity.

## Budget spreadsheets for annual trainings

Training budgets included costs of attendee and facilitator per diems and refreshments only. Costs for curriculum development, materials for delivering trainings (e.g., stationary), transportation, and venue costs were not included.

## Human resources salary spreadsheets

Salary spreadsheets reported monthly salaries for 2018, which we converted to annual salaries.

# Foreign exchange rate and inflation adjustments

We converted all costs to 2019 USD using the following rates from Table 2. All costs in Malawi kwacha (MWK) were first converted to United States dollars (USD) using the foreign exchange rate from the year in which costs were incurred.^[[1]](#footnote-1)^ All costs were then adjusted for inflation from the year in which they were incurred to 2019.^[[2]](#footnote-2)^

*Table 2. Currency conversion rates*

| **Foreign exchange rates** | |
| --- | --- |
| Foreign exchange rate 2016 | 615 MWK = 1 USD |
| Foreign exchange rate 2017 | 715 MWK = 1 USD |
| Foreign exchange rate 2018 | 725 MWK = 1 USD |
| **Inflation adjustments** |  |
| Inflation adjustment | 1 USD in 2016 = 1.06 USD in 2019 |
| Inflation adjustment | 1 USD in 2017 = 1.04 USD in 2019 |
| Inflation adjustment | 1 USD in 2018 = 1.02 USD in 2019 |

# Summary calculation methods

## Costs to establish services

Costs to establish services were generated as a sum of total transaction costs for capital hardware and capital software. Sums of total transaction costs are presented in the summary sheets for each EHS in Supplemental Files 5-11.

## Annual operations and maintenance costs

Annual operations and maintenance costs comprised capital maintenance, recurrent training, consumables, personnel, direct support, and contracted services.

**Recurrent training**

We used annual budgets for safety trainings for recurrent training costs. Safety trainings were relevant to four EHS: hygiene, PPE, cleaning, and waste management. While curricula for safety trainings covered all EHS simultaneously, we include the full training costs under each EHS, as each EHS required annual training and costs would not be substantially reduced by removing irrelevant curricula.

**Personnel and direct support**

Personnel costs were adjusted based on the percent effort dedicated to each EHS (e.g., clinic aides spent an estimated 35% of their working hours per week cleaning, so we allocated 35% of their annual salary to cleaning costs), using the weights provided in Table 3. Personnel costs for drivers for waste management were calculated in terms of hours per week transporting waste rather than percent effort. Hourly wages were calculated by dividing annual salaries by 251 working days per year and 8 working hours per day.

For annual direct support costs, we added 6% effort for supervisors, procurement officers, and logistics officers involved in EHS delivery. This cost was divided proportionally across all EHS, with EHS requiring substantial supervision of personnel (i.e., cleaning and waste management) weighed by a factor of two, i.e., percent effort for water, sanitation, hygiene, PPE, vector control, and laundry: 0.05%, percent effort for waste management and cleaning: 0.1%

Table 3. Percent efforts of staff involved in cleaning and waste management

| **Cleaning** | | |
| --- | --- | --- |
| Staff | Role | % effort of all duties |
| Nurses | Cleaning specialized equipment  Supervision of cleaning | 5% |
| Laboratory and pharmacy technicians | Cleaning specialized equipment  Autoclave operation (lab techs only)  Supervision of cleaning | 5% |
| Clinic aides | General cleaning of clinical, pharmacy, and non-clinical spaces  Initial processing of equipment for autoclaving | 35% |
| Laboratory cleaners | General cleaning of laboratory spaces  Cleaning of glassware and laboratory supplies  Autoclave operation | 70% |
| **Waste management** | | |
| Staff | Role | % effort of all duties |
| Nurses | Waste collection and segregation at point of care, Supervision of waste management | 2% |
| Laboratory and pharmacy technicians | Waste collection and segregation in laboratory and pharmacy  Supervision of waste management | 2% |
| Clinic aides | Waste transportation within-facilities  Storage and autoclave processing | 15% |
| Laboratory cleaners | Waste collection in the laboratory  Waste transportation within-facilities | 30% |
| Drivers | Transportation of waste between facilities | 2,311 hours of driver time per year |

**Capital maintenance, consumables, contracted services**

Capital maintenance, consumables, and contracted services costs across all departmental records were weighted based on the number of months of data coverage to calculate average annual costs (Table 4).

Table 4. Weights used to annualized costs of operations and maintenance expenses.

| **Weights to annualize cost and quantities for operations and maintenance expenses** | | |
| --- | --- | --- |
| **Data source** | **Months of available data** | **Weight** |
| Administrative Department | Dec 16 - May 2018 | 0.667 |
| General Stores Department | Jan 16 - Dec 17 | 0.5 |
| Pharmacy Department | Oct 17 - June 18 | 1.333 |
| Laboratory Department | Dec 16 - May 2018 | 0.667 |

Waste management had additional costs associated with vehicle operations and maintenance for transport, which we estimated using a method described by Crocker et al.^[[3]](#footnote-3)^ Briefly, we used the street addresses of facilities in the UNC Project Malawi network to calculate distances between facilities where waste was generated and stored, using open source mapping software.^[[4]](#footnote-4)^ Total distances traveled were per year were calculated based on distances between facilities reported frequencies of waste transportation trips, as reported in interviews with the head nurse. Capital maintenance costs for waste transportation vehicles were calculated based on American Automobile Association rates of USD 0.813 per mile for maintenance and tire costs, with 8284.73 miles driven per year. These costs include retail parts and labor for routine maintenance specified by the vehicle manufacturer, a comprehensive warranty, repairs to wear-and-tear items that require service during five years of operation, and one set of replacement tires.^[[5]](#footnote-5)^ Consumables for waste transportation fuel were calculated based on fuel economy rates for a 2013 Toyota Hilux,^[[6]](#footnote-6)^ annual miles driven, and fuel prices at the time of study adjusted for inflation to 2019.

**Annualized costs for capital hardware**

We classified cleaning tools and waste bins as capital hardware, but found that these items were purchased frequently, suggesting that they may also be considered consumables costs. For these items, we also calculated an annualized cost, using the weights provided in Table 4. In Supplemental Information files 5-11 presented detailed costs per EHS, summary sheets for total costs to establish services use unweighted values, as a simple sum of all observed expenses. Capital hardware tabs also present annualized costs.

1. <https://www.xe.com/currencyconverter/convert/?Amount=1&From=USD&To=MWK> [↑](#footnote-ref-1)
2. <https://fred.stlouisfed.org/series/FPCPITOTLZGUSA> [↑](#footnote-ref-2)
3. Crocker, J., et al., *The true costs of participatory sanitation: Evidence from community-led total sanitation studies in Ghana and Ethiopia.* Sci Total Environ, 2017. **601-602**: p. 1075-1083. [↑](#footnote-ref-3)
4. Open street map. <https://www.openstreetmap.org/#map=4/38.01/-95.84> [↑](#footnote-ref-4)
5. American Automobile Association. (2018) How much does it cost to drive? https://exchange.aaa.com/automotive/driving-costs/. [↑](#footnote-ref-5)
6. Fuelly. (2020). http://www.fuelly.com/car/toyota/hilux/2013 [↑](#footnote-ref-6)
