# Supplementary material for: Development and application of tools to cost the delivery of environmental health services in healthcare facilities: a financial analysis in urban Malawi
Source: BMC Health Serv Res. 2021 Apr 13;21:329. doi: 10.1186/s12913-021-06325-3 (PMC8042714; doi:10.1186/s12913-021-06325-3)
Supplement: Supplementary file 4 — Additional file 4. EHS costing frameworks [file 12913_2021_6325_MOESM4_ESM.docx]

# Comparing observed versus expected costs

| Green text = costs for which data are available and judged to be complete  Orange text = costs for which data are partially available but judged to be incomplete  Grey text = goods or services not provided at study sites  Black text = costs missing from available records | Blue fill = all costs covered under contracted services |
| --- | --- |

# Water—piped into facility

Referenced documents: [[1-4](#_ENREF_1)]

| Essential outputs | Capital hardware | Capital software | Capital maintenance | Recurrent training | Consumables | Personnel | Direct support | Financing |
| --- | --- | --- | --- | --- | --- | --- | --- | --- |
| Water source – delivery to facility | Source construction (e.g., borehole drilling) and/or municipal water main connection  Pump infrastructure | Site assessment, engineering/architectural design, and planning for water source installation | Water source and/or water main maintenance and repairs  Pump maintenance and repairs | Water source operation and testing training | Water testing and treatment supplies  Water utility bills | Staff for water testing and treatment | Water safety planning^1^ | Interest on loans for water source installation |
| Water distribution within facility | Distribution pipes  Connection points for water using infrastructure (e.g., sinks, laundry machines) | Engineering/architectural design and planning for sink and piped network installation | Distribution pipes and connection point maintenance and repairs | Water safety and testing training | Water testing and treatment supplies  Water utility bills | Staff for water testing and treatment | Water safety planning | Interest on loans for piped network installation |
| Water access at point of care | Sinks/basins with connection to piped network | Engineering/architectural design and planning for sinks | Sink maintenance and repairs | Water safety and testing training | Water testing and treatment supplies  Water utility bills | Staff for water testing and treatment | Water safety planning | Interest on loans for sink installation |
| Water access for drinking | Sink with connection to piped network and/or  Water coolers | Engineering/architectural design and planning for sinks | Sink and distribution pipe maintenance and repairs  Water cooler maintenance and repairs | Water safety and testing training | Water testing and treatment supplies  Water utility bills  Refill jugs for water coolers  Disposable cups | Staff for water testing and treatment  Staff for water cooler restocking^2^ | Water safety planning | Interest on loans for sink network installation |
| Emergency storage | Large storage containers (e.g. roof container or water tower) with connection to piped network | Site assessment, engineering/architectural design, and planning for emergency storage installation | Storage container maintenance and repairs  Distribution pipes maintenance and repairs | Water safety and testing training | Water testing and treatment supplies  Container cleaning and restocking supplies  Water utility bills | Staff for water storage monitoring, flushing, and refilling | Water safety planning | Interest on loans for storage infrastructure installation |
| ^1^Includes water quality testing, audits of water source safety. For a full guide, see <https://www.who.int/water_sanitation_health/water-quality/safety-planning/wsp-publications/en/>  ^2^Staff time for restocking water coolers considered to be negligible and excluded | | | | | | | | |

# Sanitation – non-sewered

Referenced guidelines: [[1](#_ENREF_1), [2](#_ENREF_2), [5-7](#_ENREF_5)]

| Essential outputs | Capital hardware | Capital software | Capital maintenance | Recurrent training | Consumables | Personnel | Direct support | Financing |
| --- | --- | --- | --- | --- | --- | --- | --- | --- |
| Basic Sanitation Facilities^1^ | Improved toilet  Handrails, raised seats and other disability accessibility supports  Septic tank and connecting pipes | Site assessment, engineering/architectural design, and planning for toilet installation | Toilet maintenance and repairs  Septic tank maintenance and repairs  Pit/septic tank emptying services | n/a | Anal cleansing materials  Utility costs for toilet operation | Staff for restocking anal cleansing materials^3^ | Sanitary inspections | Interest on loans for toilet installation |
| Menstrual hygiene management facilities^2^ | Private washing area with disposal bins with lids | Site assessment, engineering/architectural design, and planning for menstrual hygiene facility installation | Menstrual hygiene facility maintenance and repairs | n/a | Soap  Drying materials  Menstrual products (e.g., sanitary pads)  Utility costs for washing area | Staff for restocking soap and drying materials^3^  Staff for disposing menstrual products^4^ | Sanitary inspections | Interest on loans for washing area installation |
| ^1^ WHO guideline for number of toilets is one per 20 users for inpatient settings; at least four toilets per outpatient setting (one for staff, and for patients: one for  females, one for males and one for children) [[1](#_ENREF_1)]  ^2^ Menstrual hygiene management facilities may be included in sanitation facilities for women or as a separate facility  ^3^Staff time for restocking anal cleansing materials, soap, and drying materials considered to be negligible and excluded  ^4^Staff time for disposing menstrual products included under waste management | | | | | | | | |

# Sanitation –sewered

Referenced guidelines: [[1](#_ENREF_1), [2](#_ENREF_2), [5-7](#_ENREF_5)]

| Essential outputs | Capital hardware | Capital software | Capital maintenance | Recurrent training | Consumables | Personnel | Direct support | Financing |
| --- | --- | --- | --- | --- | --- | --- | --- | --- |
| Basic Sanitation Facilities^1^ | Improved toilet  Handrails, raised seats and other disability accessibility supports  Distribution pipes connecting to sewer mains and water source | Site assessment, engineering/architectural design, and planning for toilet installation and sewer connection | Toilet maintenance and repairs  Sewer pipe maintenance and repairs | n/a | Anal cleansing materials  Utility costs for toilet operation | Staff for restocking anal cleansing materials^3^ | Sanitary inspections | Interest on loans for toilet installation |
| Menstrual hygiene management facilities^2^ | Private washing area with disposal bins with lids | Site assessment, engineering/architectural design, and planning for menstrual hygiene facility installation | Menstrual hygiene facility maintenance and repairs | n/a | Soap  Drying materials  Menstrual products (e.g., sanitary pads)  Utility costs for washing area | Staff for restocking soap and drying materials^3^  Staff for disposing menstrual products^4^ | Sanitary inspections | Interest on loans for washing area installation |
| ^1^ WHO guideline for number of toilets is one per 20 users for inpatient settings; at least four toilets per outpatient setting (one for staff, and for patients: one for  females, one for males and one for children) [[1](#_ENREF_1)]  ^2^ Menstrual hygiene management facilities may be included in sanitation facilities for women or as a separate facility  ^3^Staff time for restocking anal cleansing materials, soap, and drying materials considered to be negligible and excluded  ^4^Staff time for disposing menstrual products included under waste management | | | | | | | | |

# Hygiene

Referenced guidelines: [[1](#_ENREF_1), [8-12](#_ENREF_8)]

| Essential outputs | Capital hardware | Capital software | Capital maintenance | Recurrent training | Consumables | Personnel | Direct support | Financing |
| --- | --- | --- | --- | --- | --- | --- | --- | --- |
| Hand hygiene at the point of care | Sink or other handwashing facility  Dispensers for alcohol-based hand rub  Disposal bins for hand drying materials | Site assessment, engineering/architectural design, and planning for sinks  Orientation on hand rub formulation and restocking | Sink maintenance and repairs  Hand rub dispenser maintenance and repair | Training on proper handwashing technique and handwashing promotion | Soap  Alcohol-based hand rub  Hand drying materials | Staff for restocking alcohol-based hand rub^1^  Staff for restocking soap and hand^1^ drying materials  Staff for disposing and/or recycling hand drying materials^2^ | Monitoring and inspections of handwashing compliance  Infection prevention and control planning | Interest on loans for sink installation |
| Handwashing facilities at sanitation facilities | Sink or other handwashing facility  Disposal bins for hand drying materials | Site assessment, engineering/architectural design, and planning for sinks | Sink maintenance and repairs | Hand hygiene promotion materials at defecation site | Soap  Hand drying materials | Staff for restocking soap and hand drying materials^1^  Staff for disposing and/or recycling hand drying materials^2^ | Monitoring and inspections of hand hygiene compliance | Interest on loans for sink installation |
| ^1^Staff time for restocking soap, alcohol-based hand rub, and hand drying materials considered to be negligible and excluded  ^2^Staff time for disposal of hand drying materials included under waste management costs | | | | | | | | |

# Personal protective equipment at the point of care

Referenced guidelines: [[9](#_ENREF_9), [12](#_ENREF_12), [13](#_ENREF_13)]

| Essential outputs | Capital hardware | Capital software | Capital maintenance | Recurrent training | Consumables | Personnel | Direct support | Financing |
| --- | --- | --- | --- | --- | --- | --- | --- | --- |
| PPE at point of care | Reusable PPE (e.g., heavy-duty aprons, protective boots) | n/a | n/a | Training on proper PPE use | Disposable PPE (single-use gloves, masks, aprons) | Staff for decontaminating, sterilization, and restocking of soiled reusable PPE^1^ | Monitoring and inspections of PPE compliance  Infection prevention and control planning | n/a |
| ^1^Staff time for restocking PPE included as part of cleaning process | | | | | | | | |

# Waste management

Referenced guidelines: [[1](#_ENREF_1), [12](#_ENREF_12), [14-16](#_ENREF_14)]

| Essential outputs | Capital hardware | Capital software | Capital maintenance | Recurrent training | Consumables | Personnel | Direct support | Financing |
| --- | --- | --- | --- | --- | --- | --- | --- | --- |
| Collection, segregation, packaging, and storage | Point-of-use waste receptacles  Interim bulk storage container  Syringe/needle cutters  Storage area refrigeration units  Reusable chemical-resistant PPE (gloves, aprons, masks)  Waste weighing scale | Orientation for waste segregation and packing procedures  Site assessment, engineering/architectural design, and planning for storage area design | Maintenance and repairs to storage infrastructure  Repairs to refrigeration units | Waste segregation and safe handling training | Disposable waste containers (e.g., sharps bins, biohazard bags)  Waste labeling materials  Disposable PPE (single-use gloves, masks, aprons)  Utility costs for refrigeration units | Staff for waste collection and packing | Safety monitoring and inspections  Immunizations for waste handlers | Interest on loans for storage site installation |
| Transportation:  pre- and post-treatment | Trolleys, carts, or other equipment for transport within facility  Transportation containers Transportation vehicles for off-site transport  Reusable chemical-resistant PPE (gloves, aprons, masks) | Orientation for transportation equipment and vehicle operation  Vehicle registration and licensing | Maintenance and repairs to transportation equipment  Vehicle repairs and maintenance | Sharps and hazardous waste safe handling and disposal training  Safe transport training | Disposable waste containers  Waste labeling materials  Disposable PPE (single-use gloves, masks, aprons)  Vehicle fuel | Staff for waste loading and unloading  Driver time | Vehicle insurance  Safety monitoring and inspections  Immunizations for waste handlers | Interest on loans for transportation vehicle and equipment |
| Pre-storage treatment:  autoclave | Autoclave machine and related supplies (e.g., autoclave trays)  Reusable chemical-resistant PPE (gloves, aprons, masks) | Autoclave procurement and installation costs  Orientation to autoclave operation and safety | Autoclave maintenance and repairs | Sharps and hazardous waste safe handling and disposal training | Utilities for autoclave operation  Waste labeling materials  Waste repackaging materials  Disposable PPE (single-use gloves, masks, aprons) | Staff for autoclave operation  Staff time for waste repackaging | Safety monitoring and inspections  Immunizations for waste handlers | Interest on loans for treatment equipment |
| Final treatment: incineration | Incinerator  waste shredders; pollution control on incinerators  Reusable chemical-resistant PPE (gloves, aprons, masks, respirators) | Site assessment, engineering/architectural design, and planning for incinerator  Incinerator licensing | Treatment equipment repairs and maintenance | Sharps and hazardous waste safe handling and disposal training | Disposable waste containers  Disposable PPE (single-use gloves, masks, aprons)  Fuel costs for incinerator | Staff for incinerator operation | Incineration air quality emissions testing  Safety monitoring and inspections  Immunizations for waste handlers | Interest on loans for capital hardware and land for treatment plant |
| Final disposal:  solid waste landfilling | Land costs  Landfill infrastructure (e.g., pit linings)  Pit digging equipment  On-site waste transport equipment (e.g., trucks, trolleys, etc.) | Site assessment, engineering/architectural design, and planning for landfill site  Landfill permits and licensing | Digging equipment maintenance and repair  Transport equipment maintenance and repair | Sharps and hazardous waste safe handling and disposal training | Disposable PPE (single-use gloves, masks, aprons)  Fuel for digging equipment | Staff for waste processing | Landfill licensing; compliance monitoring and inspections; immunizations for waste handlers | Taxes on land for landfill site  Interest on loans for capital hardware and land for disposal site |

# Cleaning

Referenced guidelines: [[1](#_ENREF_1), [17-19](#_ENREF_17)]

| Essential outputs | Capital hardware | Capital software | Capital maintenance | Recurrent training | Consumables | Personnel | Direct support | Financing |
| --- | --- | --- | --- | --- | --- | --- | --- | --- |
| General Cleaning (Low level, high level, floors, walls, and doors) | Wet cleaning tools (buckets, mops, cloths)  Dry cleaning tools (brooms, dustpans, cloths)  Reusable chemical-resistant PPE (gloves, aprons) | Orientation to cleaning protocols for newly hired staff | n/a | Refresher trainings on cleaning protocols and safety | Disposable PPE (single-use gloves, masks, aprons)  Disposable cloths  Cleaning chemicals (detergents, soaps) | Staff time for cleaning | Safety monitoring and inspections  Immunizations for cleaners | n/a |
| Cleaning infectious waste/blood spills | Wet cleaning tools (buckets, mops, cloths)  Dry cleaning tools (brooms, dustpans, cloths)  Reusable chemical-resistant PPE (gloves, aprons, face shields) | Orientation to cleaning protocols for newly hired staff  Orientation to hazardous waste safety for newly hired staff | n/a | Refresher trainings on cleaning protocols  Refresher trainings on hazardous waste safety | Disposable PPE (single-use gloves, masks, aprons)  Disposable cloths, absorbent material  Cleaning chemicals (detergents, soaps, antiseptics) | Staff time for cleaning | Safety monitoring and inspections  Immunizations for cleaners | n/a |
| Cleaning surfaces with patient contact (e.g., mattresses, delivery beds) | Wet cleaning tools (buckets, mops, cloths)  Dry cleaning tools (brooms, dustpans, cloths)  Reusable chemical-resistant PPE (gloves, aprons) | Orientation to cleaning protocols for newly hired staff  Orientation to hazardous waste safety for newly hired staff | n/a | Refresher trainings on cleaning protocols  Refresher trainings on hazardous waste safety | Disposable PPE (single-use gloves, masks, aprons)  Disposable cloths, absorbent material  Cleaning chemicals (detergents, soaps, antiseptics) | Staff time for cleaning | Safety monitoring and inspections  Immunizations for cleaners | n/a |
| Medical device/equipment disinfection and sterilization | Washing and decontamination area with waste water disposal (e.g., sluice room)  Sterilization equipment (steam sterilizer, low temp sterilizers, or chemical sterilizers)  Reusable chemical-resistant PPE (gloves, aprons) | Orientation to cleaning protocols for newly hired staff  Orientation to hazardous waste safety for newly hired staff  Site assessment, engineering/architectural design, and planning for decontamination area design  Sterilization equipment procurement and installation costs  Orientation to sterilization equipment operation and safety | Waste water disposal area maintenance and repairs  Sterilization equipment maintenance and repairs | Refresher trainings on cleaning protocols  Refresher trainings on hazardous waste safety | Disposable PPE (single-use gloves, masks, aprons)  Cleaning chemicals (detergents, soaps, antiseptics)  Packaging and wrapping for medical equipment  Labeling materials  Utilities for decontamination and sterilization equipment operation | Staff time for device packing & initial decontamination Staff time for sterilization equipment operation  Staff time for device repacking and restocking | Safety monitoring and inspections  Immunizations for cleaners | Interest on loans for washing area installation  Interest on loans for sterilization equipment |
| Cleaning toilets and handwash basins | Wet cleaning tools (buckets, mops, cloths)  Dry cleaning tools (brooms, dustpans, cloths)  Reusable chemical-resistant PPE (gloves, aprons, face shields) | Orientation to cleaning protocols for newly hired staff  Orientation to hazardous waste safety for newly hired staff | n/a | Refresher trainings on cleaning protocols  Refresher trainings on hazardous waste safety | Disposable PPE (single-use gloves, masks, aprons)  Disposable cloths, absorbent material  Cleaning chemicals (detergents, soaps, antiseptics) | Staff time for cleaning | Safety monitoring and inspections  Immunizations for cleaners | n/a |

# Laundry

|  | Capital hardware | Capital software | Capital maintenance | Recurrent training | Consumables | Personnel | Direct support | Financing |
| --- | --- | --- | --- | --- | --- | --- | --- | --- |
| Collection and packing of soiled items^1^ | Bins and cart for laundry collection and transport | Orientation to laundry protocols for newly hired staff  Orientation to hazardous waste safety for newly hired staff | Cart maintenance and repairs | Refresher trainings on cleaning protocols  Refresher trainings on hazardous waste safety | Leak-proof bags for laundry packing; Labeling materials | Staff for collecting and packaging soiled items | Safety monitoring and inspections  Immunizations for laundry handlers | n/a |
| Washing and repacking soiled items | Washing machine or physical washing area  Dryer or covered drying area | Site assessment, engineering/architectural design, and planning for laundry area  Orientation to laundry protocols for newly hired staff  Orientation to hazardous waste safety for newly hired staff | Washing machine maintenance and repairs  Dryer maintenance and repairs | Refresher trainings on cleaning protocols  Refresher trainings on hazardous waste safety | Leak-proof bags for laundry packing; Labeling materials;  Detergent; chlorine powder or concentrated chlorine  Utilities for laundry machine operation | Staff for operating washing machine and processing drying  Staff for packing and redistributing clean laundry | Safety monitoring and inspections  Immunizations for laundry handlers | Interest on loans for washing and drying machines |
| ^1^Soiled items may include bed linens, clothes for wrapping mothers and babies after delivery, staff uniforms, or reusable PPE (e.g., scrubs or surgical gowns) | | | | | | | | |

Referenced guidelines: [[1](#_ENREF_1), [12](#_ENREF_12)]

# Vector control

Referenced guidelines: [[1](#_ENREF_1)]

|  | Capital hardware | Capital software | Capital maintenance | Recurrent training | Consumables | Personnel | Direct support | Financing |
| --- | --- | --- | --- | --- | --- | --- | --- | --- |
| Insect control | Insecticide-treated bed nets for inpatient beds  Fumigation equipment (e.g., sprayers) | Orientation to fumigation equipment and protocols | Washing and reimpregnation of bed nets with insecticide  Fumigation equipment maintenance and repair | Refresher trainings on fumigation protocols  Chemical safety and poisoning prevention training | Fumigation chemicals  Insecticides^1^ | Staff time for fumigation | Safety monitoring and inspections  Immunizations for insect control workers | Interest on loans for fumigation equipment |
| Rodent and other non-insect pest control | Animal traps | Orientation to pest control equipment and protocols | Trap maintenance and repair | Refresher trainings on pest control protocols  Chemical safety and poisoning prevention training | Pest poisons | Staff time for pest control activities | Safety monitoring and inspections  Immunizations for pest control workers | n/a |
| ^1^Insect control expenses for fumigation provided as contracted serviced, but some costs were recorded for purchase of insecticides | | | | | | | | |

# References

1. Adams J, Bartram J, Chartier Y. Essential environmental health standards in health care. Geneva: World Health Organization; 2008.

2. WHO/UNICEF. Water and sanitation for health facility improvement tool (WASH FIT). Geneva: WHO; 2017.

3. WHO/UNICEF. Water, sanitation and hygiene in health care facilities: practical steps to achieve universal access to quality care. Geneva: WHO; 2019.

4. WHO/UNICEF. Expert Group Meeting on Monitoring WASH in Health Care Facilities in the Sustainable Development Goals. Geneva: 2016.

5. WHO/UNICEF. Core questions and indicators for monitoring WASH in healthcare facilities in the Sustainable Development Goals 2018. Available from: https://washdata.org/monitoring/health-care-facilities.

6. World Health Organization. Guidelines on sanitation and health. Geneva: World Health Organization, 2018.

7. WHO. Sanitary inspections for sanitation systems 2020. Available from: https://[www.who.int/water_sanitation_health/sanitation-waste/sanitation/sanitary-inspections-for-sanitation-systems/en/](http://www.who.int/water_sanitation_health/sanitation-waste/sanitation/sanitary-inspections-for-sanitation-systems/en/).

8. World health Organization. Hand hygiene self-assessment framework 2010 2010 [cited 2020 25 June]. Available from: https://[www.who.int/gpsc/country_work/hhsa_framework_October_2010.pdf](http://www.who.int/gpsc/country_work/hhsa_framework_October_2010.pdf).

9. WHO. Standard precautions in healthcare. Geneva: WHO, 2007.

10. Boyce JM, Pittet D. Guideline for hand hygiene in health-care settings: Recommendations of the healthcare infection control practices advisory committee and the hicpac/shea/apic/idsa hand hygiene task force. Am J Infect Control. 2002;30(8):S1-S46.

11. World Health Organization. Guidelines on hand hygiene in health care. Geneva: World Health Organization; 2009.

12. Centers for Disease Control and Prevention. Guidelines for Environmental Infection Prevention in Health-Care Facilities. Atlanta: CDC; 2003.

13. Ducel G, Fabry J, Nicolle L. Prevention of hospital-acquired infections: a practical guide. Geneva: 2002.

14. World Health Organization. Safe management of wastes from health care activities: a summary. Geneva: WHO; 2017.

15. United Nations Environment Program Division of technology, industry, and economics. Compendium of Technologies for Treatment / Destruction of Healthcare Waste. Osaka: UNEP; 2012.

16. Chartier Y. Safe management of wastes from health-care activities: World Health Organization; 2014.

17. CDC and ICAN. Best practices for environmental cleaning in healthcare facilities in resource-limited settings. Atlanta, GA: US Department of Health and Human Services, CDC; 2019.

18. Collaborative TS. Teach Clean Package London, UK: London School of Hygiene and Tropical Medicine; 2018. Available from: https://[www.lshtm.ac.uk/research/centres/march-centre/soapbox-collaborative/teach-clean](http://www.lshtm.ac.uk/research/centres/march-centre/soapbox-collaborative/teach-clean).

19. WHO and PAHO. Decontamination and reprocessing of medical devices for health-care facilities. Geneva: World Health Organization; 2016.
